# Short-Term Effects of Low-Level Red-Light Therapy on Central Retinal Function: A Combined Pattern ERG and Photopic ERG Study

**DOI:** 10.3390/vision10020026

**Published:** 2026-05-08

**Authors:** Muhammad Qasim, Paulo Fernandes, Jorge Jorge

**Affiliations:** Physics Center for Universities of Minho and Porto (CF-UM-UP), School of Sciences, Gualtar Campus, University of Minho, 4710-057 Braga, Portugal; jorge@fisica.uminho.pt

**Keywords:** pattern ERG, photopic 3.0, electroretinogram, red light therapy, myopes, non-myopes, correlation, axial length, choroidal thickness

## Abstract

**Background:** Low-level red-light therapy (RLRT) has emerged as a potential therapy in myopia management. However, its effects on retinal structure and function following repeated exposure remain incompletely understood. **Purpose:** To evaluate the short-term effects of RLRT on central retinal function in myopes and non-myopes using retinal electrophysiology and structural parameters. **Methods:** Thirty-six subjects underwent RLRT exposures. Retinal function was assessed using pattern electroretinography (PERG) and photopic full-field electroretinography (Photopic 3.0 ERG). Structural measurements, including axial length (AL), obtained with an optical biometer and choroidal thickness (CT), were measured using MOPTIM 3000. Outcomes were compared between baseline and post-RLRT conditions (single 3 min and single 1 min exposure), across refractive groups, and correlated with AL and CT. This study was conducted at the University of Minho in accordance with the Declaration of Helsinki. **Results:** In myopic subjects, PERG N35–P50 amplitude significantly increased after 3 min of RLRT (baseline:0.88 ± 0.21 µV; post: 1.40 ± 0.25 µV; *p* = 0.013), whereas no significant changes were observed in non-myopes (baseline: 1.32 ± 0.19 µV; post: 1.39 ± 0.21 µV; *p* = 0.47). Latencies remained stable across all groups (*p* > 0.05). No significant correlations were found between PERG and AL (ρ = −0.18, *p* = 0.44) or CT (ρ = 0.12, *p* = 0.52). Photopic 3.0 ERG showed an increase in b-wave amplitude after 3 min of RLRT in myopes (Δ = +2.42 µV, *p* = 0.06), but not in non-myopes. In myopes, AL was negatively correlated with post-therapy latency (ρ = −0.624, *p* = 0.060), while CT showed strong correlations with photopic a-wave responses after 1 min RLRT (CT vs. latency: ρ = −0.873, *p* = 0.010; CT vs. amplitude: ρ = 0.821, *p* = 0.034). **Conclusions:** Short-term exposure to RLRT, particularly at 3 min, enhances PERG and Photopic ERG responses in myopic subjects, suggesting transient improvements in retinal function with differential effects between refractive groups.

## 1. Introduction

Electroretinography (ERG) remains a fundamental electrophysiological technique for assessing retinal function in both human and animal models. Among the diverse types of electroretinograms, the pattern electroretinogram (PERG) and the light-adapted flash response, or Photopic 3.0 ERG, are widely used for both clinical and research purposes [1,2]. Standardized by the International Society for Clinical Electrophysiology of Vision (ISCEV) [3,4], these techniques evaluate distinct components of retinal function. PERG is particularly sensitive to macular function and primarily reflects the activity of ganglion cells and inner retina, whereas the Photopic 3.0 ERG evaluates cone-driven receptor responses and post-receptor processing.

Myopia, particularly high myopia, represents an increasing global health concern. Functional retinal alterations associated with myopia may precede detectable structural changes and can be effectively identified using advanced electrophysiological techniques. In this context, low-level red-light therapy (RLRT) has emerged as a novel intervention for myopia control. RLRT involves exposure to monochromatic red light (650 ± 10 nm), commonly up to 3 min per exposure, twice daily, often administered as a home-based therapy over an extended period in children and young adults [5,6,7,8]. In contrast, photobiomodulation therapy, such as Valeda Light therapy used for dry macular degeneration, employs a polychromatic light (590 to 850 nm) with a longer exposure duration, and is typically delivered under clinical supervision [9,10]. Despite the increasing clinical adoption of RLRT, current evidence is largely limited to standard protocols employing 3 min exposure durations. To date, no studies have systematically evaluated the effects of shorter exposure times [5,6,7,8,9,10]. Therefore, this study aims to investigate the short-term effects of RLRT on retinal function, as assessed by PERG and Photopic 3.0 ERG, in myopic and non-myopic individuals. In particular, we compared responses following 1 min and 3 min RLRT exposure to determine whether shorter treatment durations may produce measurable functional changes, thereby informing potential optimization of therapeutic protocols.

### 1.1. Pattern Electroretinogram (PERG)

PERG is a non-invasive electrophysiological technique that provides objective measures of the macula and retinal ganglion cells (RGCs) function. By contrast-reversing patterns (typically checkerboards or gratings), which stimulate the central retina without changing its overall luminance, PERG is particularly sensitive to inner retinal activity and central retinal processing [11,12,13,14]. The PERG waveform comprises two main components: P50, reflecting macular and pre-ganglionic activity, and N95, which is predominantly associated with RGC function. Alterations in these components are considered early indicators of inner retinal dysfunction, often preceding detectable structural changes.

RLRT has been proposed to modulate retinal function through photobiomodulation mechanisms, including the enhancement of mitochondrial activity, increased cytochrome c oxidase efficiency, and improved retinal and choroidal perfusion. Such effects may transiently influence RGC responsiveness, making PERG a suitable tool to detect short-term functional changes following RLRT exposure [9,10].

Importantly, emerging evidence in photobiomodulation suggests that retinal responses may follow a dose–response relationship, in which both insufficient and excessive exposure may result in suboptimal effects, while an intermediate “therapeutic window” maximizes its functional benefit. In this context, exposure duration represents a critical parameter that may influence the magnitude and direction of electrophysiological responses. This study assessed the PERG implicit time and amplitude to look for the central retinal function of myopic and non-myopic eyes exposed to a short-term 3 min and/or 1 min duration of RLRT. The study analyzed within-subject and between-subject effects, which gives us an insight into the impact of RLRT based on exposure duration. Further, it also investigated the cumulative effect in response to therapeutic intervention.

### 1.2. Photopic 3.0 Electroretinogram (ERG)

The photopic 3.0 ERG is a light-adapted full-field response that evaluates cone-mediated retinal activity and post-receptoral processing. The waveform consists of an initial negative a-wave, reflecting cone photoreceptor activity, followed by a positive b-wave generated primarily by bipolar and Müller cells [13,15,16].

Photopic 3.0 ERG responses are measured as amplitude (μV) and implicit time (ms) [17]. It is found that it often reduces the amplitude and delays ERG responses in high myopia [18]. This reflects compromised cone and bipolar cell function, possibly due to chorioretinal thinning, perfusion deficits, or mechanical stress. In non-myopic eyes, both PERG and photopic ERG exhibit robust, reproducible responses. P50 and N95 amplitudes are within normative limits, and a- and b-waves demonstrate full amplitudes with normal implicit times [2,13,19,20].

Mild myopia often shows minimal or no detectable changes in ERG responses. Functional losses become more apparent with increased axial elongation beyond −6.00 D [15]. Severe myopia is associated with lower PERG N95 amplitude, delayed b-wave in Photopic ERG, and region-specific dysfunction depending on choroidal thinning or fundus changes. These changes align with thinning in RNFL, GCC, and choroid assessed in OCT [13].

Combining ERG with OCT provides a multimodal approach to understanding myopic retinal changes. For instance, PERG N95 reduction correlates with ganglion cell complex thinning, enhancing diagnostic precision [18]. Normative data variability, due to age, pupil size, and stimulus conditions, in optical defocus can influence stimulus visibility, especially in PERG [3]. Emerging evidence suggests that RLRT may influence outer and middle retinal function through photobiomodulatory effects, including improved cellular metabolism and vascular responses. Given the dependence of photopic ERG responses on cone and bipolar cell integrity, this technique provides a complementary measure to PERG in evaluating the functional impact of RLRT.

From a mechanistic perspective, photobiomodulation effects are also expected to exhibit dose dependency, where exposure duration may modulate retinal responsiveness through metabolic and vascular pathways. Shorter exposures may induce subthreshold or transient effects, whereas longer exposures may enhance or stabilize functional responses, although excessive stimulation could potentially attenuate benefits.

In the present study, photopic 3.0 ERG is used alongside PERG to investigate short-term retinal responses to RLRT in myopic and non-myopic individuals, with particular focus on comparing 1 min and 3 min exposure durations as part of a dose–response framework.

## 2. Materials and Methods

This was a prospective exploratory intervention study conducted at the Clinical & Experimental Optometry Research Lab (CEORLab), University of Minho, Portugal, between February and May 2025. A single cohort of 20 young adults (10 myopes and 10 non-myopes) was enrolled and assessed under two RLRT exposure conditions. Participants were initially enrolled to undergo 3 min of RLRT exposure. The same participants were scheduled to undergo the 1 min RLRT condition. However, 4 individuals (3 myopic and 1 non-myopic) did not complete this session, resulting in a reduced sample of 16 participants (7 myopic, 9 non-myopic) for the 1 min exposure. The study adhered to the Declaration of Helsinki and received ethical approval from the Committee of the School of Sciences (CEICVS-034/2023). Written informed consent was obtained from all participants.

Eligibility criteria: Inclusion criteria comprised individuals aged 18–39 years, spherical equivalent ≥ −0.75 D for the myopia group and within ≤0.50 D for the non-myopia group, and best-corrected visual acuity ≥ 0.1 LogMAR. Participants were required to have no history of ocular or systemic disease affecting retinal function. Exclusion criteria included previous ocular surgery, systemic conditions with ocular impact, current use of ocular medication, or inability to undergo electrophysiological testing (e.g., epilepsy). Gender and age distribution were monitored to ensure a balanced group composition.

Experimental Procedures: All participants underwent baseline assessments, including visual acuity (LogMAR) and objective refraction using open-field autorefractometry (WAM-5500, Grand Seiko Co., Ltd., Hiroshima, Japan). Structural parameters included axial length (AL), measured with an optical biometer (IOL Master 500, Carl Zeiss Meditec AG, Jena, Germany), and choroidal thickness (CT), obtained using optical coherence tomography (MOPTIM 3000) (Shenzhen Certainn Technology Co., Ltd., Shenzhen, China). Retinal function was assessed using Pattern ERG and Photopic 3.0 ERG (RETI-Port/Scan21, Ronald Consults, Weisbaden, Germany). Following baseline measurements, participants received two RLRT sessions using an Eyerising Myproclear Myopia Management Device (650 ± 10 nm) (Eyerising International Pty Ltd., Melbourne, Victoria, Australia), with exposure duration determined by the cohort (1 min or 3 min). The two sessions were separated by a 30 min interval, and the fellow eye was occluded during treatment.

Electrophysiology acquisition: Electrophysiological recordings were performed in accordance with the International Society for Clinical Electrophysiology of Vision standards. To preserve physiological conditions consistent with RLRT delivery, pupils were not pharmacologically dilated. Participants underwent light adaptation (15–20 min) prior to testing. Recordings were obtained using Dawson-Trick-Litzkow (DTL-plus) (DTL Spes Medica S.r.l. Genova, Italy) fiber electrodes with reference electrodes placed near the lateral canthus, and a ground electrode at the forehead. Standardized fixation and alignment procedures were applied to ensure consistency and minimize artifacts.

Recording Conditions in PERG and Photopic 3.0 ERG: PERG responses were elicited using contrast-reversing checkerboard under photopic conditions and performed with the subject placed at 1 m from the screen projector, with the optical correction worn by all subjects, while the other eye, not being tested, was occluded. Photopic 3.0 ERG responses were recorded using standard flash stimuli (3.0 cd/m^2^). Subjects were instructed to focus on a central red fixation point and discouraged from blinking unless the test allowed them to do so.

Outcome Measures: Primary electrophysiological outcomes included PERG implicit time (N35, P50, and N95) and amplitudes (N35–P50 and P50–N95), as well as Photopic ERG a-wave and b-wave implicit times and amplitudes recorded in milliseconds (ms) and amplitudes in microvolts (μV). Structural parameters (AL and CT) were analyzed as potential correlates of functional response.

Statistical analysis: Descriptive statistics are presented as means and standard deviations. Normality of data was assessed using a Shapiro–Wilk test. When assumptions for parametric testing were not met, equivalent non-parametric analyses were performed. Intra-subject changes across time points (baseline, post-1st RRLT, post-2nd RRLT) were analyzed using repeated-measures ANOVA. The association between electrophysiological outcomes and structural parameters (axial length and choroidal thickness) was assessed using Pearson’s correlation or Spearman’s correlation coefficient, depending on data distribution. Effect sizes and 95% confidence intervals were reported where appropriate in the results. Statistical significance was set at *p* < 0.05 and analyzed using JASP Software (version 0.96.0.0. University of Amsterdam, Netherland). We presented results here in the figure forms for better understanding, however, numerical values can be seen in the supplementary data attached with the article in Tables S1–S8.

## 3. Results

Baseline characteristics of the study participants are summarized in Table 1. The mean age ranged from 24.4 to 26.3 years across groups. Myopic participants exhibited a mean spherical equivalent of −2.50 D and longer axial lengths (24.49 mm in 3 min RLRT and 24.66 mm in 1 min RLRT) compared to non-myopes (23.42 mm in 3 min RLRT and 23.91 mm in 1 min RLRT). Choroidal thickness was relatively comparable between groups, ranging from 367.6 to 376.7 µm, with similar visual acuity across groups and exposure conditions.

**Figure 1:**
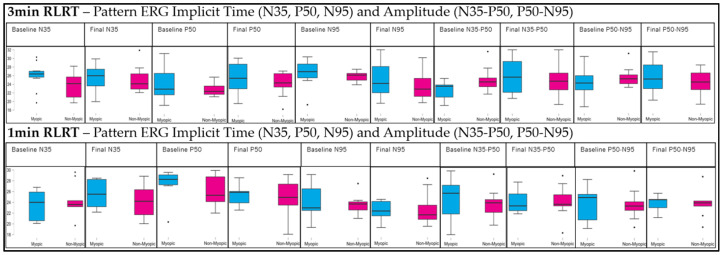
Pattern ERG implicit times and amplitudes before and after 3 min, 1 min RLRT.

Figure 1 shows the PERG implicit times (N35, P50, and N95) and amplitudes (N35–P50 and P50–N95) at baseline and following RLRT for both the 3 min and 1 min RLRT exposure conditions in myopic and non-myopic participants.

Implicit Time: No significant main effects were observed for any time component in either RLRT duration (all *p* > 0.05), indicating that implicit times remained relatively stable across baseline and post-second RLRT measurements. Similarly, no significant group effects were found (all *p* > 0.05), suggesting comparable responses between myopic and non-myopic participants. The time × group interaction effects were also non-significant for all components, indicating that changes over time did not differ between refractive groups.

Amplitude: For the 3 min RLRT condition, a significant within-subject effect was observed for both N35–P50 (*p* = 0.013) and P50–N95 (*p* = 0.009), indicating changes in amplitude across time points. However, no significant main group effects were detected (*p* > 0.05), suggesting comparable amplitude responses between myopes and non-myopes. The absence of time × group interaction further indicates that the pattern of change over time was comparable between groups. In contrast, for the 1 min RLRT, neither N35–P50 nor P50–N95 demonstrated significant exposure conditions (first session/second session), group, or interaction effects (all *p* > 0.05), reflecting stable amplitude responses across sessions in both refractive groups.

**Figure 2:**
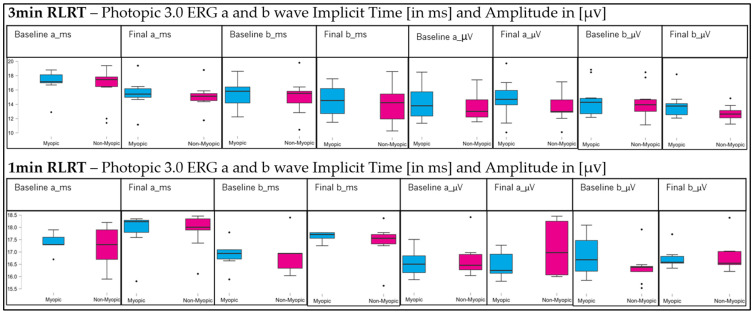
Photopic ERG implicit time and amplitude of a- and b-waves before and after RLRT.

Figure 2 shows the Photopic 3.0 ERG implicit times (a- and b-waves in milliseconds-ms) and amplitudes (a- and b-waves in microvolts-µV) following RLRT (post-first and post-second sessions) for both the 3 min and 1 min exposure conditions in myopic and non-myopic participants.

Implicit Time: For the 3 min RLRT condition, no significant main effect of time was observed for either the a-wave (*p* = 0.380) or b-wave (*p* = 0.114). Similarly, no significant group differences were found (*p* > 0.05), indicating comparable implicit times between myopes and non-myopes. However, a significant time × group interaction for the 3 min RLRT condition was detected for the b-wave (*p* = 0.041), suggesting that the temporal response pattern differed between refractive groups despite the absence of an overall time effect. For the 1 min RLRT condition, no significant main effects (time or group) or interaction effects were observed for either response wave component (all *p* > 0.05), indicating stable implicit times across sessions in both groups.

Amplitude: Under the 3 min RLRT condition, no statistically significant main effects of time were observed for either a-wave (*p* = 0.061) or b-wave (*p* = 0.739). The a-wave showed a borderline time effect (*p* = 0.061), and no significant group effects were found for either component (*p* > 0.05). The time × group interaction was non-significant for the a-wave (*p* = 0.735), while the b-wave showed a borderline interaction effect (*p* = 0.054), suggesting a possible trend toward differential amplitude response between myopes and non-myopes, though not statistically conclusive. For the 1 min RLRT condition, no significant main or interaction effects were observed for either the a-wave or b-wave amplitudes (all *p* > 0.05), indicating stable responses across sessions in both refractive groups.

**Figure 3:**
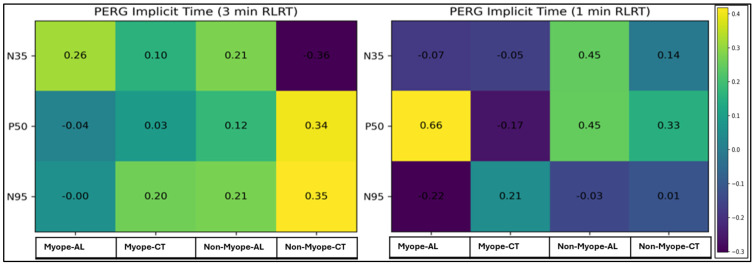
Correlation of PERG implicit time, AL, and CT following 3 min and 1 min RLRT. R−value is shown within cells and colored according to the direction of correlation.

Figure 3 presents the correlation analysis between PERG implicit time components (N35, P50, and N95) and AL, as well as CT following 3 min and 1 min RLRT in the myopic and non-myopic groups. Pearson’s and Spearman’s rho correlation coefficients were applied as appropriate based on data distribution. Overall, most correlations were weak and statistically non-significant across both RLRT exposure conditions. However, a strong and statistically significant positive correlation was observed between the P50 implicit time and the AL in the myopic participants under the 1 min RLRT condition. In contrast, correlations observed in non-myopes were generally weak and inconsistent across conditions, indicating no clear relationship between implicit time and structural parameters.

**Figure 4:**
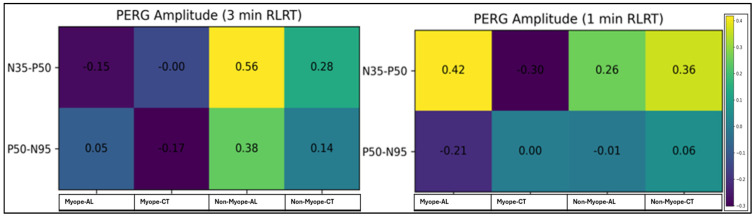
Correlation of PERG amplitude, AL, and CT following 3 min and 1 min RLRT. R−value is shown within cells and colored according to the direction of correlation.

Figure 4 presents correlations between PERG amplitude components (N35–P50 and P50–N95) and AL, as well as CT following 3 min and 1 min RLRT in myopic and non-myopic participants. Correlation coefficients, significance levels, confidence intervals, and effect sizes are reported. Overall, most correlations were weak to moderate and did not reach statistical significance across both refractive groups and exposure durations. A moderate positive association between N35–P50 amplitude and AL was observed in myopic participants under the 1 min RLRT condition. However, this did not reach statistical significance. In non-myopic participants, correlations were generally weak and variable, with no consistent patterns identified.

**Figure 5:**
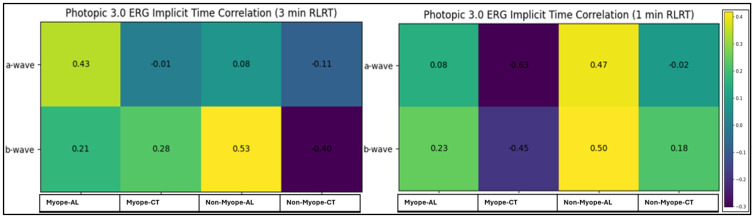
Correlation of Photopic 3.0 ERG implicit time, AL, and CT following RLRT. R-value is shown within cells and colored according to the direction of correlation.

Figure 5 presents the correlation analysis between Photopic 3.0 ERG implicit time wave components and structural parameters, including AL and CT in myopic and non-myopic eyes following 3 min and 1 min RLRT. Overall, most correlations were weak to moderate and did not reach statistical significance across exposure durations and refractive groups, indicating a limited association between photopic ERG implicit times and structural measures. However, a significant negative correlation was observed between the a-wave implicit time and CT in myopic participants under 1 min RLRT conditions, suggesting a potential association between outer retinal response and CT after short-duration RLRT.

**Figure 6:**
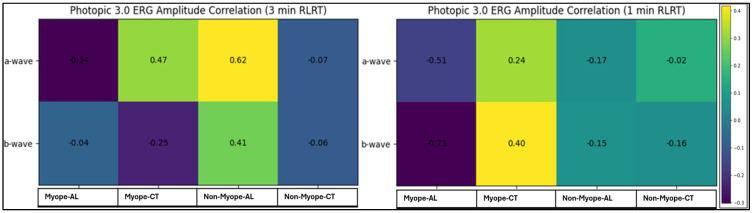
Correlation of Photopic 3.0 ERG amplitude, AL, and CT following RLRT. R−value is shown within cells and colored according to the direction of correlation.

Figure 6 shows the correlations between Photopic 3.0 ERG amplitude wave component parameters (a-wave and b-wave), AL, and CT in myopic and non-myopic participants following 3 min and 1 min RLRT. In myopic participants, a significant positive correlation was observed between the a-wave amplitude and the CT under 3 min RLRT conditions, indicating that greater choroidal thickness was associated with higher a-wave amplitudes. Additionally, a strong negative correlation was observed between b-wave amplitudes and AL under 1 min RLRT conditions, suggesting reduced b-wave amplitudes with increasing axial length. In contrast, correlations in non-myopes were generally weak and did not reach statistical significance across exposure conditions.

## 4. Discussion

This study evaluated the short-term effects of RLRT on retinal function in myopic and non-myopic eyes using PERG and Photopic 3.0 ERG metrics. The findings indicate that PERG amplitude responses are modulated over time following the 3 min RLRT protocol, whereas implicit times remain largely stable. In contrast, photopic ERG responses exhibit minimal changes.

Pattern ERG: Consistent with previous evidence that the inner retinal responses are sensitive to physiological modulation [10,14], the significant time-dependent effects observed in PERG amplitudes suggest that ganglion cells and inner retinal activity can be transiently influenced by repeated low-intensity stimulation. Specifically, the N35 –P50 and P50–N95 components demonstrate significant within-subject changes following the 3 min RLRT protocol (*p* < 0.001), supporting the sensitivity of PERG amplitude as a marker of functional retinal responsiveness. These findings are in line with prior studies showing that PERG amplitude is a sensitive indicator of retinal ganglion cell functional adaptability [14]. Despite these within-session changes, PERG implicit times remained unchanged across both exposure durations, indicating that RLRT may preferentially modulate response magnitude rather than temporal dynamics in signal processing. The absence of significant time × group interactions suggests that the observed amplitude modulations were driven primarily by the RLRT exposure itself, rather than differing refractive profiles. This supports the interpretation that short-term RLRT effects are the primary function rather than structurally mediated, at least within the timeframe assessed. No significant changes were observed under the 1 min RLRT condition, suggesting that a shorter exposure duration may be insufficient to induce measurable electrophysiological modulation. Within the dose-response framework, these findings are consistent with the presence of a minimum effective exposure threshold required to elicit detectable changes in retinal function.

This study also investigated the relationship between electrophysiological parameters (PERG and Photopic 3.0 ERG) and structural biomarkers (AL and CT) following short-term RLRT in myopic and non-myopic eyes. Overall, correlations between PERG measures (implicit time and amplitude) and structural parameters (AL and CT) were predominantly weak and not statistically significant across both exposure conditions. These findings suggest limited structure–function coupling following short-term RLRT exposure. In the 3 min RLRT condition, significant within-subject effects were observed for PERG amplitude components (N35–P50 time *p* = 0.013; P50 –N95 time *p* = 0.009), indicating transient functional modulation. A significant time × group interaction (*p* = 0.001) was also identified for N35–P50 components, suggesting that the temporal pattern of response may differ between myopic and non-myopic participants. However, the absence of significance between-group effects (all group *p* > 0.05) and non-significant post hoc comparisons at individual time-points (Pbonf > 0.05) indicates that these differences should be interpreted with caution. During the 1 min RLRT, no significant effects were detected for either Photopic 3.0 ERG or PERG (*p* > 0.05), reinforcing the notion that shorter exposure durations may be insufficient to induce measurable functional changes.

A significant finding was the strong positive correlation between P50 implicit time and AL in myopic eyes under the 1 min RLRT condition. The P50 component reflects macular and retinal ganglion cell function and is sensitive to inner retinal dysfunction [21]. The observed increase in P50 latency in eyes with greater axial elongation may indicate altered synaptic transmission, retinal stretching, or metabolic constraints associated with myopic remodeling. Structural changes in myopia, including posterior pole stretching and retinal thinning, have been well described [22,23] and may contribute to variability in electrophysiological responses.

Previous studies have reported functional retinal impairment in high myopia, including PERG amplitude reduction and latency delays [24]. However, in the present study, most structural–functional correlations were not statistically significant. This suggests that short-term RLRT exposure may not immediately modify electrophysiological–biometric relationships, or that global parameters, such as axial length, are insufficiently sensitive to detect localized neurofunctional changes.

CT did not show consistent associations with PERG measures. Although choroids play a critical role in ocular growth regulation and retinal metabolic support [25], it is a dynamic structure influenced by circadian rhythms and systemic factors. Although RLRT has been associated with axial shortening and choroidal thickening over time [26], short-duration exposure may not induce stable structural changes to influence the inner retinal electrophysiological responses.

From a mechanistic perspective, RLRT is believed to act through photobiomodulation, including enhancing mitochondrial activity, increasing ATP production, and improving cellular metabolism [27,28]. These effects are likely cumulative and may require repeated or sustained exposure before translating into measurable functional or structural adaptations. The more apparent correlations in myopic eyes may reflect increased structural susceptibility and metabolic demand in elongated eyes [5,6,7,8,22,29], potentially rendering them more responsive to photobiomodulatory effects. However, given the limited number of significant correlations and their lack of consistency across parameters and exposure conditions, these findings should be interpreted cautiously.

Overall, the present results indicate that brief RLRT exposure does not result in robust immediate coupling between structural biomarkers and PERG parameters. Nevertheless, the sensitivity of P50 latency to axial elongation in myopic eyes may warrant further investigation in studies with larger samples and longitudinal designs.

Photopic 3.0 ERG response revealed largely stable implicit times and amplitudes across therapy sessions, suggesting the limited involvement of the outer retinal photoreceptor and ON-bipolar pathways following short-term RLRT exposure. Although a significant time × group interaction was observed for b-wave implicit time under the 3 min RLRT condition, the absence of a consistent main effect suggests that photopic pathways are less responsive to RLRT than the inner retinal signals captured by PERG [2]. Repeated-measures ANOVA indicated no statistically significant changes over time (all time *p* > 0.05), no significant differences between myopes and non-myopes (all group *p* > 0.05), and no significant time × group interactions (all *p* > 0.05). Post hoc Bonferroni-adjusted comparisons at each time point also showed no significant differences (Pbonf > 0.05). Overall, these findings suggest that photopic ERG responses are less sensitive to short-term RLRT than the inner retinal signals captured by PERG [2].

Photopic 3.0 ERG primarily reflects cone-driven outer retinal (a-wave) and bipolar cell-mediated inner retinal (b-wave) function, thereby providing insight into outer and middle retinal function. In the present study, correlations between implicit time and structural parameters were weak and largely non-significant across both exposure durations. However, a significant negative correlation between the 1 min a-wave implicit time and CT in myopic eyes under 1 min RLRT suggests that thicker choroids were associated with shorter photoreceptor response latency. Given the role of choroids in oxygenation and metabolic support of the outer retina [25], increased CT may enhance photoreceptor bioenergetics, thereby reducing response delay.

Associations between implicit time and AL were largely weak. Although axial elongation is associated with retinal stretching, photoreceptor distribution, and changes in the cone density [22,29], short-term RLRT exposure is unlikely to immediately change in photo-transduction timing or synaptic transmission at the photoreceptor bipolar cell level. Unlike PERG, which reflects ganglion cell function, photopic ERG is less sensitive to localized macular dysfunction and more reflective of generalized retinal activity [30].

In contrast, amplitude parameters showed relatively stronger structural associations, particularly in myopic eyes. A significant positive correlation between a-wave amplitude and CT under the 3 min RLRT exposure condition suggests that increased choroidal thickness may enhance photoreceptor response magnitude. This is consistent with the proposed photobiomodulation effects of RLRT, including improving mitochondrial function and ATP production, which may transiently augment outer retinal signal amplitudes [27,31].

Additionally, a strong negative correlation was observed between b-wave amplitude and AL in myopic eyes under the 1 min RLRT condition, indicating reduced bipolar cell-mediated responses with axial length [22]. These may reflect the impact that axial elongation has on retinal cell density and altered synaptic organization [30], potentially increasing the susceptibility of middle retinal pathways to functional modulation.

In non-myopic eyes, correlations were weak and inconsistent, suggesting relative structural and physiological stability. In contrast, myopic eyes may exhibit heightened responsiveness due to biomechanical stress, altered retinal metabolism, and choroidal thinning [27,30]. Although longer-term studies have demonstrated that RLRT induced axial shortening and choroidal thickening over time [28]. The present findings suggest that short-duration exposure primarily reveals subtle outer and middle retinal functional associations rather than robust structural–functional coupling.

Collectively, these results indicate that photopic ERG amplitude parameters may be more sensitive to structural influences than implicit times in the context of acute RLRT exposure, particularly in myopic eyes. Furthermore, the efficacy of RLRT increases as exposure increases, as reported by the recently published systematic review and meta-analysis. The subjects continued therapy for 12 months, and showed more improvements than those who continued for 6 months (i.e., +0.68 D in SER, −0.30 mm in AL, and +26.7 μm) in CT, respectively. Although exposure 7 days a week or 5 days a week gives comparable results [32], no subject reported any adverse events in our study, possibly because of the short-term duration with only two sessions. However, long-term use, up to 1 year, may reduce cone density in the paracentral foveal area as reported in a Chinese study [33]. The correlation analysis in our study provides additional insight into structure–function relationships. Although most correlations are weak, significant associations like CT and photopic responses can be given consideration in myopia management via red light therapy.

The present study has several limitations that should be considered when interpreting the findings. First, RLRT was delivered at 650 ± 10 nm wavelength of RLRT, which is under the safety limits to be used for myopia management therapy. This differs from other photobiomodulation therapies, such as Valeda therapy for dry age-related macular degeneration, which employs broader light intensity from a 550 nm to 890 nm wavelength [9,10]. As such, the present results may not be directly comparable across photobiomodulation modalities. Second, the relatively small sample size, exploratory design limits, and statistical power preclude causal inference, particularly structure–function correlations, and further studies are needed, especially for finding the structure–function correlation. Thirdly, the study population consisted of young adults (≥18 years), whereas the RLRT used in previous studies was usually conducted in children aged 5 to 16 years to manage myopia. Age-related differences in retinal plasticity and ocular growth dynamics may influence responsiveness to photobiomodulation, potentially limiting the generalizability of these findings to a younger cohort. Additionally, the short duration of RLRT and the absence of control conditions likely reflect acute physiological responses rather than cumulative therapeutic effects. CT is also subject to diurnal and systemic variability, which may influence correlation strength.

Future studies should incorporate longitudinal designs incorporating repeated RLRT exposure to determine whether cumulative treatment enhances structure–function coupling. The integration of ERG measurements with other OCT metrics (e.g., cone outer segment length, outer nuclear layer thickness, and ganglion cell–inner plexiform layer analysis) may provide more precise structure–function correlations.

## 5. Conclusions

In conclusion, short-term RLRT demonstrates a selective effect on inner retinal functions, as evidenced by increased PERG amplitudes following the 2 min exposure, without significant changes in implicit time. Shorter exposure (1 min RLR) did not produce measurable electrophysiological changes, supporting the presence of a duration-dependent response and a potential minimum effective threshold within a dose-response framework. Overall, these findings indicate that RLRT induces transient functional modulation rather than immediate structural adaptation, with differential sensitivity across retinal layers. Further longitudinal studies are required to determine whether repeated exposure leads to sustained electrophysiological and structural changes relevant to myopia management.

## Figures and Tables

**Table 1:** Baseline characteristics of the study participants.

Duration of Therapy	Category	Mean Age (yrs)	Gender		Total Subject	Logmar Visual Acuity		Spherical Equivalent (D)	Axial Length (mm)	Choroidal Thickness (µm)
			Male	Female		Unaided	Aided			
3 min RLRT	Myope	26.3	3	7	20	−0.122	−0.180	−2.52	24.49	367.6
	Non-Myope	24.6	5	5		−0.168	−0.182	0.10	23.91	370.3
1 min RLRT	Myope	25.14	2	5	16	−0.160	−0.234	−2.50	24.66	376.7
	Non-Myope	24.44	4	5		−0.169	−0.164	0.08	23.42	375.3

## Data Availability

Data available on request.

## Supplementary Materials

The following supporting information can be downloaded at: https://www.mdpi.com/article/10.3390/vision10020026/s1, Pattern ERG Data (including Table S1–S4), Table S1: Implicit Time Descriptives–PERG–3 min & 1 min RLRT–Myopes & Non-Myopes. Table S2: Amplitude Descriptives–PERG–3 min & 1 min RLRT–Myopes & Non-Myopes. Table S3: 3 min & 1 min RLRT PERG Implicit Time Correlation-AXL & CT in Myope & Non-Myope. Table S4: 3 min & 1 min RLRT PERG Amplitude Correlation-AXL & CT in Myope & Non-Myope. Photopic 3.0 ERG Data (including Table S5–S8). Table S5: Descriptive Statistics–Photopic 3.0 ERG–Implicit Time- 3 min & 1 min RLRT. Table S6: Descriptive Statistics–Photopic 3.0 ERG–Amplitude–3 min & 1 min RLRT. Table S7: Correlation–Photopic 3.0 Implicit Time with AXL & CT–3 min & 1 min RLRT. Table S8: Correlation–Photopic 3.0 Amplitude with AXL & CT–3 min & 1 min RLRT.

## Abbreviations

The following abbreviations are used in this manuscript:

RLRT	Repeated Low-Level Red-Light Therapy
PERG	Pattern Electroretinogram
AL/AL	Axial Length
CT	Choroidal Thickness
ms	Milliseconds
μV	Microvolts
DTL	Dawson, Trick, & Litzkow Electrode
