# Supplementary material for: Short-Term Effects of Low-Level Red-Light Therapy on Central Retinal Function: A Combined Pattern ERG and Photopic ERG Study
Source: Vision (Basel). 2026 May 8;10(2):26. doi: 10.3390/vision10020026 (PMC13214938; doi:10.3390/vision10020026)
Supplement: Supplementary file 1 [file vision-10-00026-s001.zip › vision-4249562-supplementary.pdf]

Table S1: Implicit Time Descriptives – PERG – 3min & 1min RLRT – Myopes & Non-Myopes

Component	PERG Measurement	Myope	Non-Myope	Time P-Value	Group P-Value	Time x Group P-Value
		Mean±SD	Mean±SD			
3min RLRT N35	Baseline	25.99±3.19	23.81±2.93	0.710	0.290	0.521
	After 1st RLRT	24.97±4.41	24.27±2.21			
	After 2nd RLRT	24.28±2.88	24.06±2.64			
3min RLRT P50	Baseline	48.92±5.58	47.22±2.24	0.287	0.502	0.622
	After 1st RLRT	47.21±4.04	47.98±5.69			
	After 2nd RLRT	46.61±3.14	45.47±2.35			
3min RLRT N95	Baseline	89.74±8.91	87.53±3.43	0.062	0.340	0.989
	After 1st RLRT	84.59±8.93	82.63±8.59			
	After 2nd RLRT	89.64±8.82	87.01±7.01			
1min RLRT N35	Baseline	23.41±2.93	24.10±3.42	0.997	0.813	0.464
	After 1st RLRT	23.94±3.90	23.64±2.28			
	After 2nd RLRT	24.36±2.34	23.11±2.58			
1min RLRT P50	Baseline	47.76±2.70	46.89±2.65	0.214	0.606	0.986
	After 1st RLRT	48.27±4.67	47.62±3.27			
	After 2nd RLRT	46.50±2.34	45.94±4.18			
1min RLRT N95	Baseline	85.50±8.95	83.87±4.57	0.334	0.829	0.661
	After 1st RLRT	84.74±8.32	87.60±8.91			
	After 2nd RLRT	88.06±5.67	88.66±9.32			

Time p-value = within-subject effect; Group p-value = between-subject effect; Time × Group p-value = interaction effect.

Table S2: Amplitude Descriptives – PERG – 3min & 1min RLRT – Myopes & Non-Myopes

Component	PERG Measurement	Myope		Non-Myope		Time P-Value	Group P-Value	Time x Group P-Value
		Mean	STD	Mean	STD			
3min RLRT N35-P50	Baseline	2.379×10^{-6}	1.065×10^{-6}	3.560×10^{-6}	1.406×10^{-6}	0.013*	0.502	0.622
	After 1st RLRT	2.670×10^{-6}	1.364×10^{-6}	2.412×10^{-6}	1.265×10^{-6}			
	After 2nd RLRT	3.220×10^{-6}	8.917×10^{-7}	3.070×10^{-6}	8.706×10^{-7}			
3min RLRT P50-N95	Baseline	4.498×10^{-6}	2.391×10^{-6}	5.500×10^{-6}	1.648×10^{-6}	0.009**	0.944	0.137
	After 1st RLRT	4.007×10^{-6}	2.155×10^{-6}	3.738×10^{-6}	2.248×10^{-6}			
	After 2nd RLRT	5.370×10^{-6}	1.642×10^{-6}	4.790×10^{-6}	1.161×10^{-6}			
1min RLRT N35-P50	Baseline	3.100×10^{-6}	7.257×10^{-7}	2.956×10^{-6}	4.851×10^{-7}	0.939	0.866	0.587
	After 1st RLRT	2.857×10^{-6}	6.852×10^{-7}	3.144×10^{-6}	6.784×10^{-7}			
	After 2nd RLRT	3.071×10^{-6}	7.761×10^{-7}	3.078×10^{-6}	1.032×10^{-6}			
1min RLRT P50-N95	Baseline	4.814×10^{-6}	1.693×10^{-6}	4.878×10^{-6}	1.516×10^{-6}	0.633	0.956	0.974
	After 1st RLRT	4.429×10^{-6}	1.591×10^{-6}	4.544×10^{-6}	8.428×10^{-7}			
	After 2nd RLRT	4.900×10^{-6}	1.328×10^{-6}	4.822×10^{-6}	2.104×10^{-6}			

*p<0.05, significant within-subject (time) effect. **p<0.05, significant time×group interaction

Table S3: 3min & 1min RLRT PERG Implicit Time Correlation - AXL & CT in Myope & Non-Myope

Implicit Time PERG Correlation with AXL & CT	Myope					Non-Myope				
	r-value	p-value	95% CI		Effect Size	r-value	p-value	95% CI		Effect Size
			Lower	Upper				Lower	Upper	
3min – N35 – AXL	0.257*	0.274	-0.209	0.628	0.263	0.213**	0.367	-0.253	0.599	0.217
3min – N35 – CT	0.100*	0.674	-0.358	0.520	0.100	-0.360**	0.119	-0.692	0.098	-0.377
3min – P50 – AXL	-0.039*	0.870	-0.473	0.411	-0.039	0.116**	0.144	-0.344	0.531	0.117
3min – P50 – CT	0.031*	0.898	-0.467	0.417	-0.031	0.339**	0.144	-0.122	0.680	0.353
3min – N95 – AXL	-4.356×10^{-4}*	0.999	-0.443	0.442	-4.356×10^{-4}	0.212**	0.369	-0.254	0.598	0.215
3min – N95 – CT	0.199*	0.399	-0.267	0.590	0.202	0.355**	0.125	-0.104	0.689	0.371
1min – N35 – AXL	-0.071**	0.810	-0.580	0.478	-0.071	0.445*	0.064	-0.028	0.755	0.478
1min – N35 – CT	-0.053**	0.857	-0.568	0.491	-0.053	0.135*	0.592	-0.354	0.567	0.136
1min – P50 – AXL	0.656**	0.011	0.192	0.880	0.785	0.448*	0.040	0.028	0.778	0.534
1min – P50 – CT	-0.170**	0.562	-0.642	0.397	-0.171	0.334*	0.175	0.157	0.693	0.348
1min – N95 – AXL	-0.218**	0.454	-0.671	0.353	-0.222	-0.030**	0.906	-0.490	0.443	-0.030
1min – N95 – CT	0.209**	0.473	-0.362	0.666	0.212	0.010**	0.967	-0.459	0.475	0.010

*Pearson's Correlation **Spearman's rho Correlation

Table S4: 3min & 1min RLRT PERG Amplitude Correlation - AXL & CT in Myope & Non-Myope

Amplitude PERG Correlation with AXL & CT	Myope					Non-Myope				
	r-value	p-value	95% CI		Effect Size	r-value	p-value	95% CI		Effect Size
			Lower	Upper				Lower	Upper	
3min – N35-P50 – AXL	-0.146*	0.540	-0.553	0.317	-0.147	0.560**	0.010	0.156	0.803	0.632
3min – N35-P50 – CT	-0.003*	0.991	-0.445	0.440	-0.003	0.278**	0.235	-0.187	0.642	0.286
3min – P50-N95 – AXL	0.049*	0.839	-0.403	0.481	0.049	0.384**	0.094	-0.070	0.707	0.405
3min – P50-N95 – CT	-0.167*	0.481	-0.568	0.297	-0.169	0.138**	0.561	-0.324	0.547	0.139
1min – N35-P50 – AXL	0.418**	0.137	--	--	0.445	0.258*	0.301	-0.238	0.647	0.264
1min – N35-P50 – CT	-0.302**	0.293	--	--	-0.312	0.362*	0.140	-0.126	0.709	0.379
1min – P50-N95 – AXL	-0.208**	0.476	-0.665	0.363	-0.211	-0.011*	0.966	-0.475	0.458	-0.011
1min – P50-N95 – CT	0.002**	0.994	-0.529	0.532	0.002	0.056*	0.825	-0.422	0.510	0.056

*Pearson's Correlation **Spearman's rho Correlation

Results – Photopic 3.0 ERG

Table S5: Descriptive Statistics – Photopic 3.0 ERG – Implicit Time- 3min & 1min RLRT

Photopic 3.0 Component	Measurements	Myope	Non-Myope	Time P-Value	Group P-value	Time x Group P-Value
		Mean±SD	Mean±SD			
3min a-wave [ms]	Baseline	17.13±1.65	16.48±2.66	0.380	0.546	0.826
	After 1st RLRT	17.47±1.11	17.42±1.10			
	After 2nd RLRT	17.51±2.02	17.15±1.72			
3min b-wave [ms]	Baseline	32.65±1.04	32.53±1.23	0.114	0.271	0.041
	After 1st RLRT	32.41±0.95	35.52±6.67			
	After 2nd RLRT	32.27±1.07	32.05±1.24			
1min a-wave [ms]	Baseline	17.39±0.38	17.24±0.77	0.098	0.704	0.724
	After 1st RLRT	16.59±1.99	15.88±1.88			
	After 2nd RLRT	16.14±2.42	16.32±1.94			
1min b-wave [ms]	Baseline	32.80±1.15	32.62±1.38	0.215	0.726	0.324
	After 1st RLRT	32.69±1.01	33.09±1.54			
	After 2nd RLRT	32.60±0.78	31.64±2.92			

*Post-hoc comparisons (Bonferroni-adjusted) between myopes and non-myopes at each time point showed no statistically significant differences (all P>0.05). Values are expressed as mean±standard deviation. Time p-value = within-subject effect; Group p-value = between-subject effect; Time × Group p-value = interaction effect.

Table S6: Descriptive Statistics – Photopic 3.0 ERG – Amplitude – 3min & 1min RLRT

Photopic 3.0 Component	Measurements	Myope	Non-Myope	Time P-Value	Group P-Value	Time x Group P-Value
		Mean±SD	Mean±SD			
3min a-wave [V]	Baseline	$(2.54\pm1.35)\times10^{-5}$	$(2.12\pm1.09)\times10^{-5}$	0.061	0.111	0.735
	After 1st RLRT	$(2.03\pm1.17)\times10^{-5}$	$(1.16\pm5.05)\times10^{-5}$			
	After 2nd RLRT	$(2.37\pm1.35)\times10^{-5}$	$(1.86\pm1.05)\times10^{-5}$			
3min b-wave [V]	Baseline	$(8.54\pm3.28)\times10^{-5}$	$(8.15\pm3.16)\times10^{-5}$	0.739	0.068	0.054
	After 1st RLRT	$(1.01\pm4.30)\times10^{-4}$	$(5.77\pm2.33)\times10^{-5}$			
	After 2nd RLRT	$(9.80\pm4.45)\times10^{-5}$	$(7.27\pm2.57)\times10^{-5}$			
1min a-wave [V]	Baseline	$(2.12\pm1.13)\times10^{-5}$	$(2.35\pm1.45)\times10^{-5}$	0.855	0.342	0.626
	After 1st RLRT	$(2.02\pm7.79)\times10^{-5}$	$(2.08\pm1.06)\times10^{-5}$			
	After 2nd RLRT	$(1.67\pm6.62)\times10^{-5}$	$(2.43\pm1.23)\times10^{-5}$			
1min b-wave [V]	Baseline	$(9.52\pm3.53)\times10^{-5}$	$(7.58\pm2.65)\times10^{-5}$	0.923	0.763	0.097
	After 1st RLRT	$(8.64\pm2.51)\times10^{-5}$	$(8.82\pm4.40)\times10^{-5}$			
	After 2nd RLRT	$(8.65\pm2.98)\times10^{-5}$	$(8.86\pm4.38)\times10^{-5}$			

*Post-hoc comparisons (Bonferroni-adjusted) between myopes and non-myopes at each time point showed no statistically significant differences (all P>0.05). Values are expressed as mean ± standard deviation. Time p-value = within-subject effect; Group p-value = between-subject effect; Time × Group p-value = interaction effect.

Table S7: Correlation – Photopic 3.0 Implicit Time with AXL & CT – 3min & 1min RLRT

Implicit Time Photopic Correlation with AXL & CT	Myope					Non-Myope				
	r-value	p-value	95% CI		Effect Size	r-value	p-value	95% CI		Effect Size
			Lower	Upper				Lower	Upper	
3min – a-wave [ms] – AXL	0.426**	0.061	-0.021	0.731	0.455	0.083**	0.729	-0.374	0.507	0.083
3min – a-wave [ms] – CT	-0.005**	0.982	-0.447	0.438	-0.005	-0.114**	0.633	-0.530	0.346	-0.114
3min – b-wave [ms] – AXL	0.214*	0.365	-0.252	0.600	0.217	0.525**	0.017	0.107	0.785	0.583
3min – b-wave [ms] – CT	0.280*	0.231	-0.185	0.643	0.288	-0.400**	0.081	-0.716	0.052	-0.423
1min – a-wave [ms] – AXL	0.077**	0.792	-0.473	0.584	0.078	0.467**	0.051	2.044×10^{-5}	0.767	0.506
1min – a-wave [ms] – CT	-0.631**	0.016	-0.870	-0.150	-0.743	-0.016**	0.951	-0.479	0.455	-0.016
1min – b-wave [ms] – AXL	0.229**	0.432	-0.344	0.677	0.233	0.496**	0.036	0.038	0.782	0.544
1min – b-wave [ms] – CT	-0.454	0.103	-0.794	0.100	-0.490	0.185**	0.463	-0.309	0.600	0.187

*Pearson's Correlation **Spearman's rho Correlation

Table S8: Correlation – Photopic 3.0 Amplitude with AXL & CT – 3min & 1min RLRT

Amplitude Photopic 3.0 ERG Correlation with AXL & CT	Myope					Non-Myope				
	r-value	p-value	95% CI		Effect Size	r-value	p-value	95% CI		Effect Size
			Lower	Upper				Lower	Upper	
3min – a-wave [V] – AXL	-0.339**	0.144	-0.679	0.122	-0.353	0.616**	0.004	0.238	0.832	0.718
3min – a-wave [V] – CT	0.465**	0.039	0.029	0.753	0.504	-0.066**	0.781	-0.494	0.388	-0.066
3min – b-wave [V] – AXL	-0.035**	0.882	-0.471	0.414	-0.035	0.412**	0.071	-0.038	0.723	0.438
3min – b-wave [V] – CT	-0.249**	0.290	-0.623	0.217	-0.254	-0.060**	0.801	-0.490	0.393	-0.060
1min – a-wave [V] – AXL	-0.508**	0.063	-0.818	0.031	-0.560	-0.167**	0.509	-0.588	0.326	-0.168
1min – a-wave [V] – CT	0.240**	0.409	-0.333	0.683	0.244	-0.019**	0.942	-0.481	0.452	-0.019
1min – b-wave [V] – AXL	-0.726**	0.003	--	--	-0.920	-0.148**	0.558	-0.575	0.343	-0.149
1min – b-wave [V] – CT	0.398**	0.160	--	--	0.421	-0.164**	0.514	-0.586	0.328	-0.166

*Pearson's Correlation **Spearman's rho Correlation
